# Characterisation of deep dorsal horn projection neurons in the spinal cord of the Phox2a::Cre mouse line

**DOI:** 10.1177/17448069221119614

**Published:** 2022-09-03

**Authors:** Éva Kókai, Wafa AA Alsulaiman, Allen C Dickie, Andrew M Bell, Luca Goffin, Masahiko Watanabe, Maria Gutierrez-Mecinas, Andrew J Todd

**Affiliations:** 1 School of Psychology and Neuroscience, College of Medical, Veterinary and Life Sciences, 3526 University of Glasgow, Glasgow, UK; 2 School of Veterinary Medicine, College of Medical, Veterinary and Life Sciences, 3526 University of Glasgow, Glasgow, UK; 3 Department of Anatomy, Hokkaido University School of Medicine, Sapporo, Japan

**Keywords:** Anterolateral system, antenna cell, peptidergic nociceptor, low-threshold mechanoreceptor, Tac1, substance P

## Abstract

Projection neurons belonging to the anterolateral system (ALS) underlie the perception of pain, skin temperature and itch. Many ALS cells are located in laminae III-V of the dorsal horn and the adjacent lateral white matter. However, relatively little is known about the excitatory synaptic input to these deep ALS cells, and therefore about their engagement with the neuronal circuitry of the region. We have used a recently developed mouse line, Phox2a::Cre, to investigate a population of deep dorsal horn ALS neurons known as “antenna cells”, which are characterised by dense innervation from peptidergic nociceptors, and to compare these with other ALS cells in the deep dorsal horn and lateral white matter. We show that these two classes differ, both in the density of excitatory synapses, and in the source of input at these synapses. Peptidergic nociceptors account for around two-thirds of the excitatory synapses on the antenna cells, but for only a small proportion of the input to the non-antenna cells. Conversely, boutons with high levels of VGLUT2, which are likely to originate mainly from glutamatergic spinal neurons, account for only ∼5% of the excitatory synapses on antenna cells, but for a much larger proportion of the input to the non-antenna cells. VGLUT1 is expressed by myelinated low-threshold mechanoreceptors and corticospinal axons, and these innervate both antenna and non-antenna cells. However, the density of VGLUT1 input to the non-antenna cells is highly variable, consistent with the view that these neurons are functionally heterogeneous.

## Introduction

The anterolateral system (ALS) consists of projection neurons in the spinal cord that transmit sensory information that is perceived as pain, skin temperature and itch. The cells of origin of the ALS are found in four main clusters, which are located in lamina I, the lateral spinal nucleus (LSN), the lateral part of the deep dorsal horn (laminae IV-V, extending into the lateral white matter) and a ventromedial region that includes parts of laminae VI-VIII and X.^1,2^ In addition, a few ALS cells are found in lamina III, and many of these have long dorsally directed dendrites that extend into the superficial dorsal horn (SDH, laminae I-II).^3^ Based on their dendritic morphology, these (together with similar cells in lamina IV) have been named antenna cells,^4,5^ and they have been shown to receive a dense excitatory synaptic input from peptidergic nociceptors, as well as numerous inhibitory synapses from interneurons that express neuropeptide Y (NPY).^6–8^

ALS neurons project to several brain regions, including the thalamus, periaqueductal grey matter, lateral parabrachial area (LPb) and various medullary nuclei. Much of their input to the brain terminates on the contralateral side, although some neurons project bilaterally.^9^ The ALS underlies perception of pain, temperature and itch, as shown by the effects of anterolateral cordotomy, which severs these axons.^10,11^ However, many of its constituent neurons also respond to innocuous mechanical stimuli.^2^ Anatomical tracing studies have shown that these different populations of ALS cells have specific termination sites within the brain.^12–14^ For example, the lamina I cells project mainly to the dorsolateral nucleus of the LPb and the posterior, posterior triangular and ventral posterolateral nuclei of the thalamus. In contrast, axons of the lateral laminae IV-V and LSN cells terminate in the internal lateral nucleus of LPb and the central lateral nucleus of the thalamus. There are also important differences in the response properties of these two populations. Lamina I ALS cells have small receptive fields, and most respond to noxious stimuli, with some being activated by innocuous mechanical stimuli, and others responding exclusively to non-noxious temperature changes.^15–19^ In contrast, deep dorsal horn cells have relatively large receptive fields, and most of these cells are described as “wide dynamic range” (WDR), responding weakly to innocuous mechanical stimuli and strongly to noxious heat or pinch; however, others respond only to innocuous or noxious cutaneous stimuli.^20–23^ Projection cells in both of these regions can also respond to itch-inducing (pruritic) stimuli.^24^

It has recently been shown that a mouse line that expresses Cre under control of the promoter for the transcription factor Phox2a can be used to reveal some of the ALS neurons belonging to each of the populations described above.^5,25^ Crossing this Phox2a::Cre line with appropriate reporter lines, results in permanent expression of fluorescent proteins in these cells, thus allowing them to be identified and labelled, without the need for retrograde tracing. The aim of this study was to use the Phox2a::Cre line to compare the anatomical features of two of these populations: the antenna cells and other (“non-antenna”) cells located in the deep dorsal horn and the adjacent part of the white matter (lateral funiculus).

## Materials and methods

All animal experiments were approved by the Ethical Review Process Applications Panel of the University of Glasgow and were performed in accordance with the UK Animals (Scientific Procedures) Act 1986.

### Animals

We used three genetically modified mouse lines during the course of this study. One was the BAC transgenic line (Phox2a::Cre), in which Cre recombinase is expressed under control of the Phox2a promoter.^5,25^ The other two were reporter lines (Ai9, Ai32), in which Cre-mediated excision of a STOP cassette drives expression of either tdTomato (Ai9) or yellow fluorescent protein (YFP) fused to channelrhodopsin (Ai32). The Phox2a::Cre line was crossed with each of these reporters to generate mice in which cells that expressed Phox2a at any point during development were permanently marked by the presence of tdTomato or YFP, with the latter being targeted to the plasma membrane.

Five Phox2a::Cre;Ai9 mice (three female, two male) and 3 Phox2a::Cre;Ai32 mice (two female, one male), weighing between 17 and 31 g were deeply anaesthetised (pentobarbitone, 20 mg i.p.) and perfused through the left cardiac ventricle with fixative containing 4% freshly depolymerised formaldehyde in phosphate buffer. Spinal cords were removed and post-fixed for 2 h at 4°C.

### Immunohistochemistry, confocal scanning and analysis

Multiple-labelling immunofluorescence reactions were performed as described previously^26^ on 60 μm thick transverse or parasagittal sections of spinal cord, which had been cut with a vibrating blade microtome (Leica VT1200 or VT1000). The sources and concentrations of antibodies used are listed in Table 1. Sections were incubated for 3 days at 4°C in primary antibodies diluted in phosphate buffered saline (PBS) that contained 0.3 m NaCl, 0.3% Triton X-100 and 5% normal donkey serum, and then overnight in appropriate species-specific secondary antibodies (Jackson Immunoresearch, West Grove, PA) that were raised in donkey and conjugated to Alexa 488, Alexa 647, Rhodamine Red or biotin. All secondary antibodies were used at 1:500 (in the same diluent), apart from those conjugated to Rhodamine Red, which were diluted to 1:100. Biotinylated secondary antibodies were detected with Pacific Blue conjugated to avidin (1:1000; Life Technologies, Paisley, UK). Following the immunocytochemical reaction, sections were mounted in anti-fade medium and stored at −20°C. Sections were scanned with Zeiss 710 LSM (Argon multi-line, 405 nm diode, 561 nm solid state and 633 nm HeNe lasers) or Zeiss 900 Airyscan (405, 488, 561, 640 nm diode lasers) confocal microscopes, using 40× or ×63 oil-immersion objectives (numerical apertures of 1.3 and 1.4, respectively). In all cases, the aperture was set to one Airy unit or less. All analyses were performed with Neurolucida for Confocal software (MBF Bioscience, Williston, VT, USA).

**Table 1.** Antibodies used in this study.

Antigen	Species	Dilution	Source	Catalogue #	RRID
mCherry^a^	Chicken	1:1000	Abcam	Ab205402	RRID:AB_2722769
mCherry^a^	Rat	1:500	Invitrogen	M11217	RRID:AB_2536611
GFP^b^	Chicken	1:1000	Abcam	Ab13970	RRID:AB_300798
CGRP	Sheep	1:2000	Enzo	BML-CA1137	RRID:AB_2243859
CGRP	Guinea pig	1:5000	Bachem	T-5027	RRID:AB_518152
CGRP	Rabbit	1:2000	Enzo	BML-CA1134	RRID:AB_2068527
Substance P	Rat	1:200	Oxford biotechnology	OBT06435	
NPY	Rabbit	1:1000	Peninsula	T-4070	RRID:AB_518504
CTb	Goat	1:5000	List biological	703	RRID:AB_10013220
VGLUT2	Guinea pig	1:5000	Millipore	ab2251	RRID:AB_1587626
Homer	Goat	1:1000	M Watanabe		RRID:AB_2631104
VGLUT1	Guinea pig	1:5000	Millipore	ab5905	RRID:AB_2301751

CGRP, calcitonin gene-related peptide; CTb, cholera toxin B subunit; GFP, green fluorescent protein; NPY, neuropeptide Y.

^a^The mCherry antibodies recognise TdTomato.

^b^The GFP antibody recognises YFP.

### Identification of different populations among Phox2a-positive neurons

To distinguish antenna cells from other types of Phox2a-positive projection neuron, we immunostained transverse sections from the L2 segments of 3 Phox2a::Cre;Ai9 mice (two female, one male), using antibodies against mCherry (which also detects tdTomato), calcitonin gene-related peptide (CGRP, sheep antibody), substance P (SP) and NPY. Between 19 and 26 sections from each animal were scanned to include the entire dorsal horn on both sides. The locations of all tdTomato-positive cells in these sections were plotted onto outlines of the spinal grey matter, and the cells were assigned to three different populations: (1) lamina I neurons, (2) antenna cells (identified by the presence of a high density of contacts from axons immunoreactive for CGRP, SP and NPY), and (3) other “non-antenna” cells, which were identified by the lack of numerous contacts from axons containing CGRP, SP or NPY. For each animal, all cell locations were then plotted onto a single outline of the dorsal horn.

To determine the proportion of Phox2a-positive antenna cells that were retrogradely labelled from LPb, and the proportion of retrogradely labelled antenna cells that were Phox2a-positive, we used transverse sections from the L2 segments of 6 Phox2a::Cre;Ai9 mice that had been used in a previous study (experiments 1–6 in reference^25^). In each case, the animals had received an injection of 300 nL 1% cholera toxin B subunit (CTb) targeted on the left LPb, and in two cases, an equivalent injection was also made into the right LPb. The sections were reacted with antibodies against mCherry, CTb, CGRP (guinea pig antibody) and NPY. Between 6 and 15 sections from each mouse were examined. For the four mice with unilateral injections of CTb, only the dorsal horn contralateral to the LPb injection was scanned and analysed, whereas both dorsal horns from the two mice that had received bilateral CTb injections were examined. All Phox2a-positive antenna cells in these sections were initially identified by the presence of a high density of contacts from CGRP- and NPY-immunoreactive boutons onto tdTomato-labelled dendrites and cell bodies in laminae III-V. The presence or absence of CTb was noted in each case. We then looked for CTb-labelled cells in these laminae that were Phox2a-negative and were associated with bundles of CGRP- and NPY-immunoreactive axons.

### Comparison of antenna and non-antenna types among deep Phox2a cells

To compare antenna cells with other Phox2a-positive cells in the deep dorsal horn or lateral white matter, we initially reconstructed cell bodies and dendritic trees of 10 cells of each type from confocal scans of sagittal sections from the L5 segment of 3 Phox2a::Cre;Ai32 mice. The Ai32 reporter line (instead of Ai9) was used for these experiments because the membrane-associated YFP expression is more suitable for neuronal reconstruction.^25^ The sections had been reacted with antibodies against green fluorescent protein (GFP, this antibody also detects YFP), CGRP (rabbit antibody), VGLUT2 and the postsynaptic density protein Homer. The cell bodies and dendritic trees (including dendritic spines) were initially drawn using Neurolucida. We next recorded the locations of Homer puncta, which indicate the presence of excitatory synapses^27^ on the reconstructed cells. Since the dendrites of these cells often had a relatively large diameter, Homer puncta on dendritic shafts that were orthogonal to the plane of section could be clearly identified, whereas those that were not (i.e., those on the medial or lateral aspect of the dendrites, which appear at the top or bottom of the dendrites when it is viewed in parasagittal section) could not always be recognised with certainty. We therefore only included those Homer puncta that were orthogonal to the section plane. This approach will inevitably have resulted in an underestimate of the absolute density of excitatory synapses on dendritic shafts, and also of the relative density on shafts compared to spines. Homer puncta were clearly visible on the great majority of dendritic spines, and these were also plotted. We then revealed the channels corresponding to VGLUT2 and CGRP and noted the presence or absence of each type of immunostaining in structures that contacted each of the Homer puncta. VGLUT2 is strongly expressed in axons originating from glutamatergic spinal neurons, and is generally weakly expressed in (or absent from) many primary afferents.^28^ We therefore distinguished between strong and weak VGLUT2-immunoreactivity in this analysis.

VGLUT1-immunoreactive boutons in the dorsal horn can originate from either myelinated low-threshold mechanoreceptors (A-LTMRs, which include tactile afferents and proprioceptors), or from corticospinal axons.^28–32^ We looked for evidence of input from VGLUT1 boutons to antenna and deep non-antenna cells, by examining sections from the L5 segments of the same 3 Phox2a::Cre;Ai32 mice. The sections were reacted with antibodies directed against GFP, CGRP (rabbit antibody), Homer and VGLUT1. Because the density of input from VGLUT1-immunoreactive boutons to the antenna cells was found to be relatively low, we restricted our analysis to parts of dendritic trees (identified by the presence of numerous contacts from CGRP-immunoreactive axons) that were nearly parallel to the plane of section, and could therefore be captured in relatively short z-series from the confocal microscope. However, for the non-antenna cells we only analysed those for which the soma was in the section, because YFP-labelled dendrites that lacked CGRP contacts could have arisen from cells in any part of the spinal cord. Since A-LTMRs arborise in a region extending from the inner part of lamina II to lamina V, for both populations we only examined dendrites within this zone. VGLUT1-immunoreactive profiles in the dorsal horn can originate from either A-LTMRs or corticospinal tract axons. However, unlike corticospinal tract boutons, those belonging to A-LTMRs frequently form multiple output synapses, and in some cases glomerular arrangements.^33–36^ We therefore distinguished between VGLUT1 boutons that were associated with only single Homer puncta (and are likely to have originated from corticospinal boutons) and those associated with more than one Homer punctum (which are likely to be A-LTMR terminals).

### Combined immunohistochemistry and fluorescent in situ hybridisation

Combined in situ hybridisation and immunohistochemistry was conducted using hybridisation chain reaction version 3.0. Buffers and a probe against the *Tac1* gene, comprising 17 oligonucleotide pairs, were purchased from Molecular Instruments (Los Angeles, USA). Lumbar spinal cord segments from 2 Phox2a::Cre;Ai9 mice (one male, one female) that had been perfused with fixative, as described above, were embedded in OCT medium and cut into 50 μm thick sagittal sections with a cryostat (Leica CM1950). Sections were mounted onto SuperFrost Plus slides (48311–703; VWR) and air dried. Sections were dehydrated in ethanol (50%, 70%, 100%, 100% for 5 min each) and washed twice in PBS. Slides were incubated in a 10 μg/mL solution of proteinase K (25530-015; Invitrogen) for 10 min at 37°C.

Hybridization and amplification were performed according to manufacturer’s instructions.^37^ The *Tac1* probe was used at a concentration of 16 nM with a B1 adaptor for 16 h at 37°C. Amplification was carried out at room temperature for 16 h in the dark with amplifier hairpins for the B1 adaptor conjugated to Alexa 647. Slides were washed two times for 15 min in ×5 saline sodium citrate containing 0.1% Tween 20 and then fixed in 4% formaldehyde for 30 min at room temperature prior to commencing slide-mounted immunofluorescence reactions. Following the fixation step, slides were washed two times for 15 min in PBS.

To identify Phox2a+ antenna cells in the sections reacted with probes against *Tac1*, we subsequently carried out immunostaining with antibodies directed against CGRP (raised in rabbit) and mCherry (raised in rat). Slides were incubated overnight at room temperature in primary antibodies diluted in PBS that contained 0.3 m NaCl, 0.3% Triton X-100 and 5% normal donkey serum. Sections were incubated in species specific secondary antibodies for 4 h at room temperature. Following the immunohistochemical reaction, sections were mounted in Prolong Glass antifade medium (ThermoFisher Scientific, Paisley, UK) and stored at −20°C. Sections were scanned with a Zeiss 710 LSM confocal microscope using the ×63 oil-immersion objective.

### Characterisation of antibodies

The sources and dilutions of primary antibodies used in the study are listed in Table 1. The mCherry and GFP antibody were raised against recombinant full-length proteins, and their distribution matched those of native tdTomato and YFP fluorescence, respectively. The sheep and rabbit CGRP antibodies were raised against a synthetic peptide corresponding to a portion of rat α-calcitonin gene-related peptide, while the guinea pig antibody was raised against the whole peptide. The distribution of staining with these antibodies closely matches that reported in the literature. The monoclonal SP antibody detects the C-terminal 5–8 amino acids of SP and does not appear to recognise neurokinin B.^38,39^ Immunostaining with the NPY antibody is abolished by pre-incubation with NPY.^40^ Specificity of the CTb antibody is shown by lack of staining in regions that did not contain retrogradely labelled neurons. The VGLUT1 and VGLUT2 antibodies were raised against 19 or 18 amino acid sequences, respectively, from the corresponding rat protein. Both stain identical structures to those detected by well-characterised rabbit antibodies against the corresponding transporter.^28^ The affinity purified Homer antibody was raised against amino acids 1–175 of mouse Homer 1 and detects a band of the appropriate size in immunoblots of mouse brain extracts.^27^

### Statistics

T-tests were used to compare the densities of dendritic spines and Homer puncta between antenna cells and deep non-antenna cells. A two-way ANOVA was used to compare the density of synapses from VGLUT1-immunoreactive boutons onto deep non-antenna cells in grey and white matter.

## Results

### Distribution of different populations of Phox2a cells

Crossing Phox2a::Cre mice with the Ai9 or Ai32 reporter resulted in the appearance of tdTomato- or YFP-positive neurons, respectively, in the spinal cord. For convenience, we refer to these as Phox2a cells.

**Figure 1.**
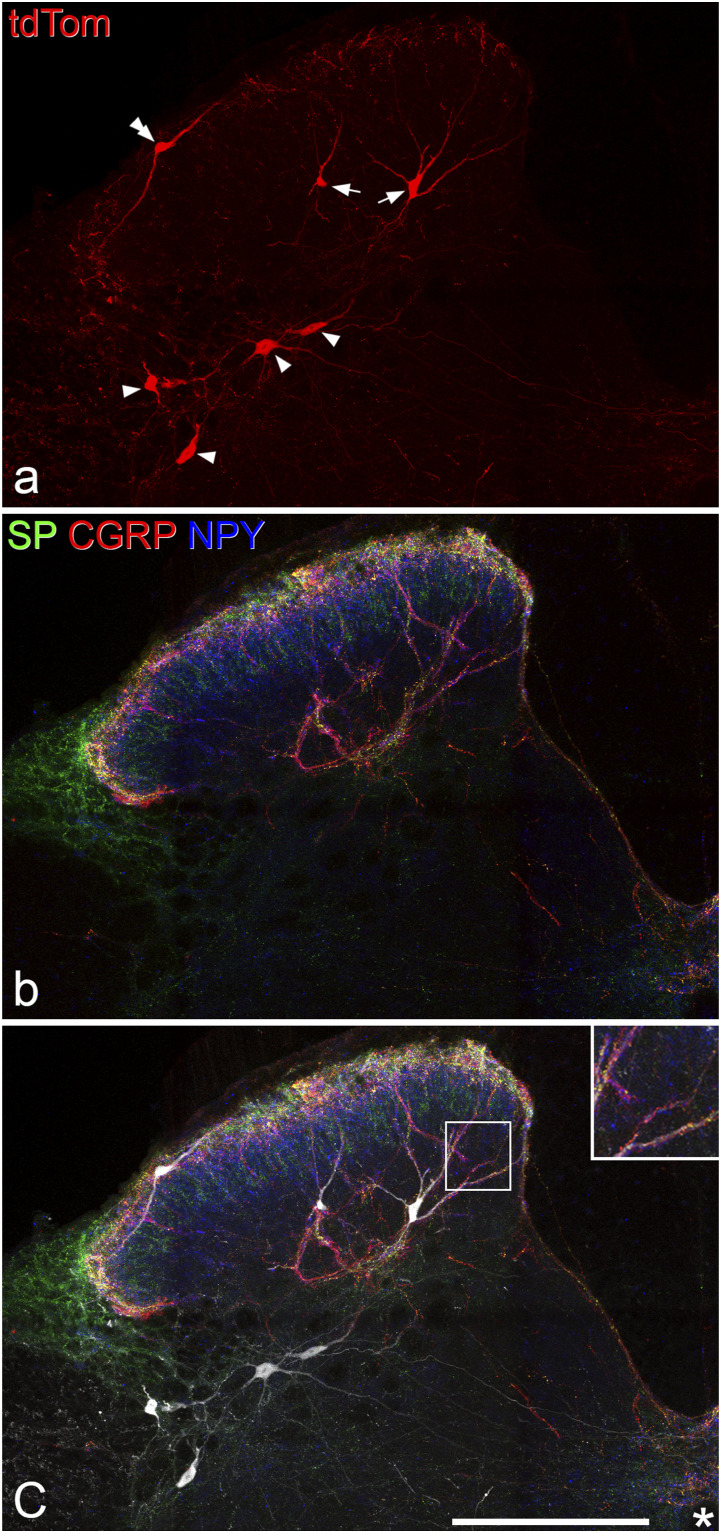
Phox2a cells and their relationship to axons immunoreactive for CGRP, SP or NPY. (a), A transverse section through the dorsal horn from the L2 segment of a Phox2a::Cre;Ai9 scanned to reveal tdTomato in Phox2a cells. Two antenna cells (arrows) are present in this section, together with four deep dorsal horn Phox2a cells that are not antenna cells (arrowheads) and a lamina I cell (double arrowhead). (b) The same section showing immunoreactivity for SP (green), CGRP (red) and NPY (blue). Axons containing these peptides are concentrated in the superficial dorsal horn, but also form bundles in deeper laminae. (c) Staining for tdTomato (grey) has been superimposed on that for the neuropeptides, revealing the association between bundles of peptidergic axons and the antenna cells. The area in the box is shown as an inset at higher magnification. All images are projections of 16 confocal optical sections at 2 μm z-spacing. Scale bar = 200 μm. CGRP, calcitonin gene-related peptide; NPY, neuropeptide Y; SP, substance P.

**Figure 2.**
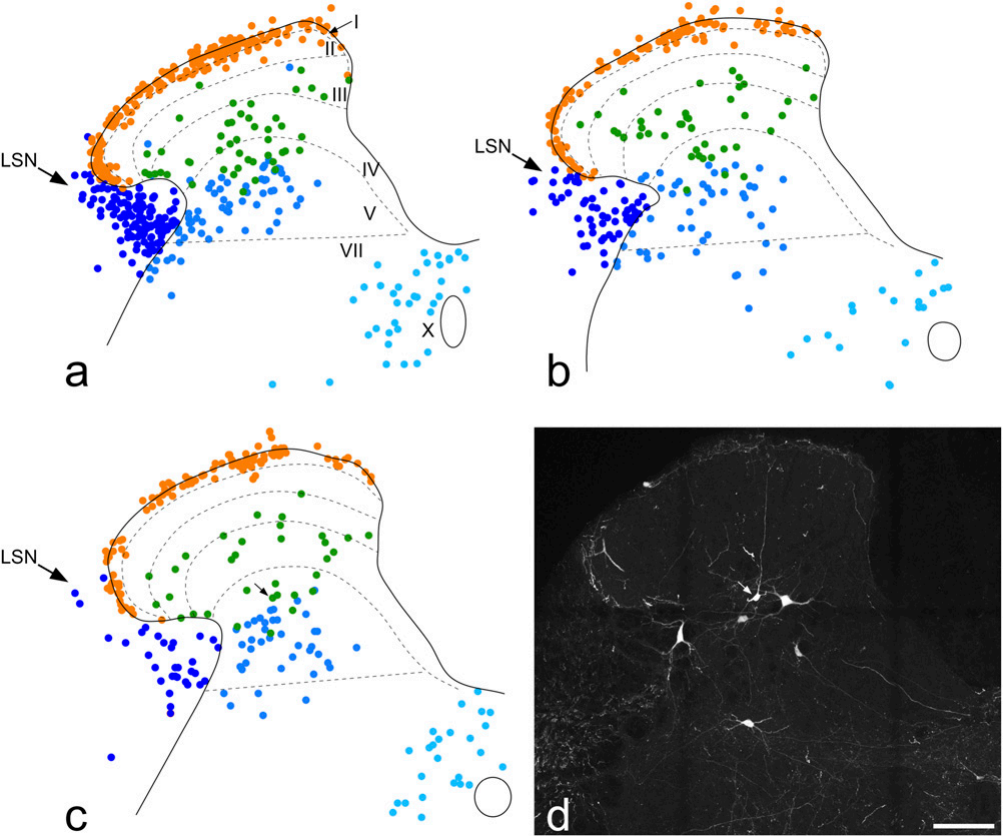
The distribution of different classes of Phox2a cells. (a–c), Plots showing the locations of Phox2a cells in the L2 segment in 3 Phox2a::Cre;Ai9 mice. All Phox2a cells (apart from those in the ventral horn) that were present on either side in 26, 23 and 19 transverse sections (respectively) were plotted onto an outline of the grey matter. Approximate laminar boundaries are shown, together with the position of the lateral spinal nucleus (LSN). Note that lamina VI is not present in the L2 spinal segment, and therefore laminae V and VII are directly adjacent. Cells were assigned to three main classes: lamina I (orange), antenna (green) and deep non-antenna (blue). The latter were subdivided into those near the central canal (light blue), those in the lateral white matter (dark blue) and the remainder (mid-blue), which were mainly present in the lateral part of lamina V but with some extension dorsally and ventrally. (d) A confocal image showing one of the sections from the animal represented in (c). A lamina V antenna cell is indicated with an arrow, and this has a dorsal dendrite that extends into the superficial dorsal horn. This cell corresponds to the one marked with the arrow in (c). The image in (d) is a projection of 17 optical sections at 2 μm z-spacing. Scale bar = 50 μm.

The distribution of Phox2a cells in the L2 spinal cord segments of Phox2a::Cre;Ai9 mice was similar to that reported previously^5,25^ (Figures 1 and 2). Within the dorsal horn these cells were concentrated in lamina I, present at a lower density in the deeper laminae of the dorsal horn (III-V) and largely absent from lamina II. Although Phox2a cells were present throughout the mediolateral extent of laminae I and III-IV, those in lamina V were largely restricted to its lateral part. Phox2a cells were also present in a region that extended from the medial part of lamina VII to the area round the central canal (lamina X). Many Phox2a cells were present in the white matter, with most being in the lateral funiculus (lateral to lamina V) and a few in the LSN.

In sections immunostained for CGRP, SP and NPY we found that many of the Phox2a cells in laminae III-IV were associated with axons that were immunoreactive for these peptides (Figure 1). Specifically, both the cell bodies and dendrites of these cells were in contact with numerous axonal boutons that contained CGRP and SP (derived from peptidergic nociceptors) and also with many boutons that contained NPY (which are likely to originate from local NPY-expressing inhibitory interneurons^8,41^). This pattern of input allowed these cells to be readily identified, and distinguished from other Phox2a cells, which had very few (if any) contacts from these peptidergic axons. A few cells with this pattern of contacts from peptidergic axons were present in the deepest part of lamina II, and some were also seen in lamina V. Altogether 121 Phox2a cells with numerous CGRP/SP and NPY contacts were seen during this analysis (Table 2). Most of these cells (84) were located in laminae II-IV, with the remaining 37 in lamina V. For the great majority of the cells located in laminae II-IV (70/84, 83%), dorsal dendrites could be followed into the SDH (Figure 1), but in some cases this was not possible, probably because these dendrites were cut during tissue sectioning. These Phox2a cells presumably correspond to the neurokinin 1 receptor (NK1r) -expressing neurons in laminae III and IV that were described in early immunohistochemical studies of rat spinal cord^42,43^ and subsequently shown to be ALS projection cells.^3,7,8,44–47^ However, in the mouse many of these cells lack the NK1r.^6^ Most of the Phox2a cells in lamina V that had numerous contacts from CGRP/SP and NPY axons had at least one dorsally directed dendrite, and in some cases (16/37, 43%) dendrites could be followed into the SDH (Figure 2(d)). For the remaining cells of this type, dendrites could not be followed into the SDH, and again, this may have resulted from transection of dendrites during tissue sectioning. We define the neurons in laminae II-V that were associated with numerous CGRP/SP and NPY axons as antenna cells*.*^4,5,48^

**Table 2.** Quantification of different classes of Phox2a cells in the L2 segment.

	Lamina I cells		Antenna cells		Other cells (lateral WM)		Other cells (III-V, lateral VII)		Other cells (medial VII, X)	
	Total	Per 10 sections	Total	Per 10 sections	Total	Per 10 sections	Total	Per 10 sections	Total	Per 10 sections
Animal A (*n* = 26 sections)	229	88.1	47	18.1	132	50.8	66	25.4	36	13.8
Animal B (*n* = 23 sections)	120	52.2	41	17.8	58	25.2	48	20.9	19	8.3
Animal C (*n* = 19 sections)	102	53.7	33	17.3	33	17.4	39	20.5	25	13.2

Many of the Phox2a cells in lamina V, some of those in laminae III-IV, as well as cells in the lateral white matter and near the central canal, received few (if any) contacts from CGRP/SP or NPY axons, and we refer to these as “non-antenna” cells. These could be subdivided into three groups: (a) cells in medial part of lamina VII and the area around the central canal (lamina X), (b) cells in laminae III-V and the lateralmost part of lamina VII, and (c) cells in the lateral white matter (Figure 2, Table 2). The anatomical separation of antenna and non-antenna cells is likely to reflect the different developmental origin of these two classes, since the antenna cells (together with lamina I Phox2a cells) are born before the deep non-antenna cells.^5^

We previously found some variation in the number of Phox2a lamina I cells between different Phox2a::Cre;Ai9 mice.^25^ A similar variability was seen for the three mice analysed in this part of the study, since the density of both lamina I cells and deep non-antenna cells in the lateral white matter was considerably higher in the mouse illustrated in Figure 2(a). However, the density of antenna cells was very similar in the three mice (Table 2).

### Retrograde labelling of antenna cells in the Phox2a::Cre line

**Figure 3.**
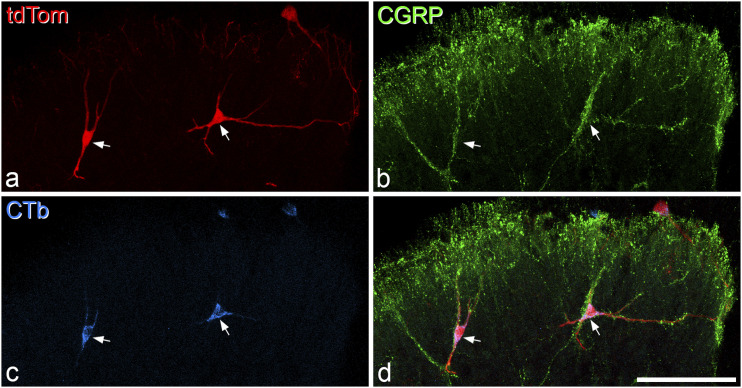
Retrograde labelling of Phox2a antenna cells. (a) Two Phox2a antenna cells (arrows) revealed by expression of tdTomato (tdTom) in a transverse section through the dorsal horn from the L2 segment of a mouse that had received an injection of cholera toxin B subunit (CTb) into the lateral parabrachial area on the opposite side. (b) immunostaining for calcitonin gene-related peptide (CGRP) shows the association of peptidergic afferent boutons with the dendrites of these two cells. (c, d) both of the Phox2a cells are retrogradely labelled with CTb (blue). Images are projections from seven optical sections at 2 μm z-spacing. Scale bar = 100 μm.

We have previously reported that in the rat, all of the antenna cells (identified by NK1r expression) can be retrogradely labelled from the caudal ventrolateral medulla, while ∼two-thirds of them can be labelled from the LPb.^3^ We therefore examined tissue from 6 Phox2a::Cre;Ai9 mice that had received injections of CTb into the LPb. We identified 60 Phox2a-positive antenna cells (defined by the presence of contacts from CGRP and NPY axons) in sections from the L2 segments of these mice (6–21 cells per animal) and found that 47 of these (78%) were retrogradely labelled with CTb (Figure 3). During this analysis we did not find any tdTomato-negative CTb-labelled cells that were associated with CGRP and NPY axons. This indicates that for those antenna cells that can be retrogradely labelled from LPb, all of them are apparently captured in the Phox2a::Cre;Ai9 cross. We also noted that many of the Phox2a-positive “non-antenna” cells in the deep dorsal horn and lateral white matter were retrogradely labelled from the LPb, although we did not quantify this.

### Excitatory synaptic inputs to antenna and non-antenna cells in deep dorsal horn

**Figure 4.**
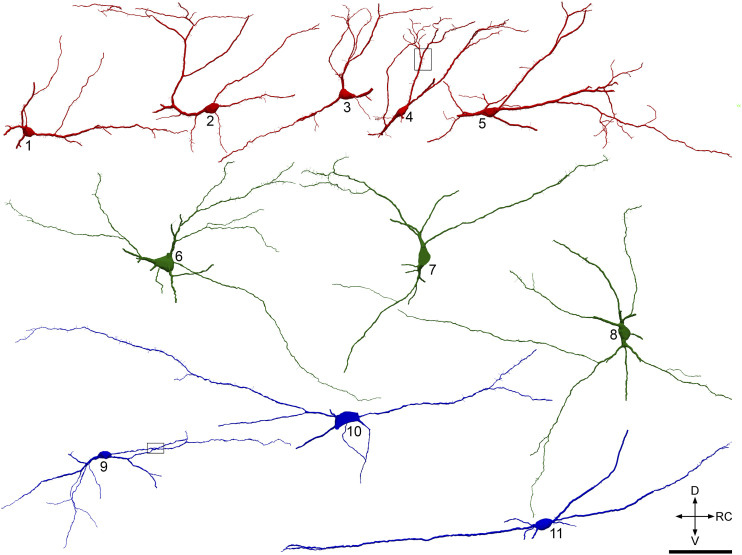
Reconstructions of deep dorsal horn Phox2a cells. Neurolucida reconstructions of antenna and non-antenna cells in sagittal sections from Phox2a::Cre;Ai32 mice. Antenna cells are shown in red, and the non-antenna cells in either green (for those located in the grey matter) or blue (for those in the white matter). The boxes on cells 4 and 9 indicate regions of these two cells that are illustrated in Figure 5. Scale bar = 100 μm. D, dorsal; V, ventral; RC, rostrocaudal.

**Figure 5.**
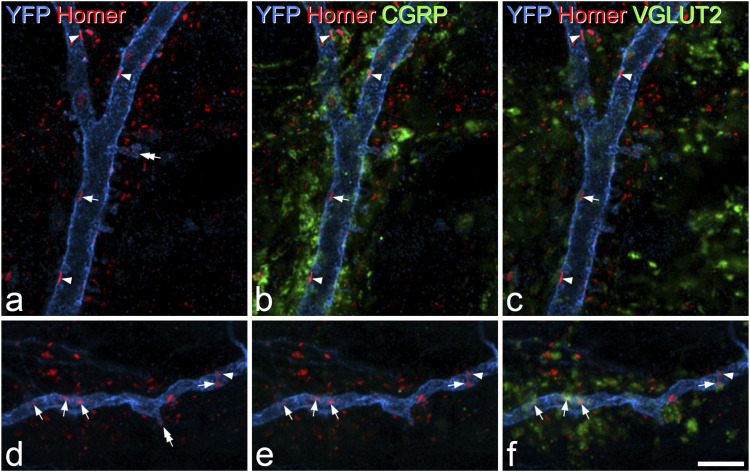
Contacts on deep dorsal horn Phox2a cells from boutons that are immunoreactive for CGRP and/or VGLUT2. (a) Part of the dorsal dendrite of an antenna cell (cell #4 in Figure 4) from a Phox2a::Cre;Ai32 mouse labelled to reveal YFP (blue) and Homer (red). Several Homer puncta can be seen on the dendritic shaft. (b, c) These images show immunostaining for either CGRP or VGLUT2 in green. Several of the Homer puncta are in contact with profiles that are CGRP-immunoreactive, and some of these also show weak VGLUT2-immunoreactivity (examples indicated with arrowheads). The arrow points to a Homer punctum that is not associated with CGRP. In this case, there is very weak VGLUT2-immunoreactivity adjacent to the Homer. The double arrow in (a) indicates a Homer punctum on a dendritic spine. (d) Part of the dendrite of a non-antenna cell that was located in the white matter (cell #9 in Figure 4) from a Phox2a::Cre;Ai32 mouse labelled to reveal YFP (blue) and Homer (red). Again, several Homer puncta can be seen on the dendritic shaft. (e, f) These images show immunostaining for either CGRP or VGLUT2 in green, although there is very little CGRP in this region. Several of the Homer puncta are in contact with VGLUT2-immunoreactive boutons (some shown with arrows). The arrowhead points to a Homer punctum that is not associated with VGLUT2. The double arrow in (d) indicates a Homer punctum on a dendritic spine. Images in (a)–(c) are from a single optical section, those in (d)–(f) are projected from three optical sections at 0.5 μm z-spacing. Scale bar: 5 μm. CGRP, calcitonin gene-related peptide; YFP, yellow fluorescent protein.

In sagittal sections from Phox2a::Cre;Ai32 mice that had been reacted to reveal YFP, Homer, CGRP and VGLUT2, we identified antenna cells in laminae III-IV by the presence of numerous contacts from CGRP-immunoreactive axons. We reconstructed 10 of these cells, together with 10 non-antenna cells, using Neurolucida software. Quantitative data are shown in Table 3 and some of these cells are illustrated in Figure 4. Six of the non-antenna cells were located in the grey matter and four in the white matter lateral to lamina V. For most cells, some of the dendrites left the section at the cut surfaces, and the reconstructions of dendritic trees are therefore incomplete. All of the cells possessed dendritic spines (Figure 4), although these were significantly more numerous on the antenna cells than on the non-antenna cells (Table 3). Homer puncta could be detected on most of the dendritic spines, and the proportion was slightly higher for the antenna cells. All of the cells possessed Homer puncta on their dendritic shafts (Figure 5). As noted above, these were only analysed when they were orthogonal to the plane of section, and this will have resulted in an underestimate of shaft synapses. With this caveat, we found that the density of shaft Homer puncta was significantly higher for the antenna cells (Table 3).

**Table 3.** Quantitative data for excitatory synapses on different classes of Phox2a cells.

	Dendrite length (μm)	Spine density (/100 μm)	% Spines with homer	Homer+ spine density (/100 μm)	Homer shaft density (/100 μm)
Antenna (*n* = 10 cells)	1201(515–1983)	14(9.6–21.9)	78.2(72.6–83.9)	11(8.49–18.4)	19.9(16–26.2)
Non-antenna (*n* = 10 cells)	1541(714–2162)	5.8(2.9–8.1)	74(67.3–81.8)	4.3(2.2–5.9)	14.2(9.5–18.8)
Significance(p)		2.5 × 10^−6^	0.04	4.3 × 10^−6^	0.003

The second and third columns show the length of dendrite analysed for each cell and the density of dendritic spines. The fourth and fifth columns show the proportion of dendritic spines that contained a Homer punctum, and the density of spines that possess a Homer punctum. The sixth column shows the density of Homer puncta on the dendritic shafts. Significance values, shown in the fourth row, were obtained from t-tests comparing values for antenna and non-antenna cells.

We then analysed contacts from boutons that were immunoreactive for CGRP and/or VGLUT2 on the Homer puncta on 6 cells of each type (Table 4). For the antenna cells, we found that 65.8% of Homer puncta were associated with CGRP-immunoreactive boutons (some of which showed weak VGLUT2). The proportions associated with CGRP-negative boutons that showed strong or weak VGLUT2 staining were 4.6 and 7.4%, respectively, while 22.1% of Homer puncta were not associated with either CGRP- or VGLUT2-immunoreactive boutons. During this analysis, we often observed VGLUT2 boutons that were in contact with the dendrites of the antenna cells, but were not associated with Homer puncta on the antenna cells themselves. In many cases these boutons contacted Homer puncta that were spatially separated from the antenna cell dendrite, suggesting that they were presynaptic to other neurons in the vicinity.

**Table 4.** Inputs from CGRP and VGLUT2 boutons to Phox2a cells.

	Number of Homer puncta analysed	% CGRP	% CGRP-negative (VGLUT2 strong)	% CGRP-negative (VGLUT2 weak)	% neither
Antenna (*n* = 6 cells)	336 (191–503)	65.8 (61.7–75.1)	4.6 (3.6–6.8)	7.4 (5.8–9.4)	22.1 (15.6–27.2)
Non-antenna (*n* = 6 cells)	310 (207–412)	4 (1.1–11.4)	38.2 (28.4–51.2)	16.3 (13.1–18.4)	41.6 (24.2–48.4)

CGRP, calcitonin gene-related peptide.

Columns 3–6 show the proportion of Homer puncta that were associated with boutons that were immunoreactive for CGRP, VGLUT2 or neither.

For non-antenna cells, only 4% of Homer puncta were associated with CGRP-immunoreactive boutons, whereas 38.2% and 16.3%, respectively, were in contact with CGRP-negative boutons that showed strong or weak VGLUT2-immunoreactivity (Table 4). The remaining 41.6% of Homer puncta on these cells were not in contact with either CGRP- or VGLUT2-immunoreactive boutons.

**Figure 6.**
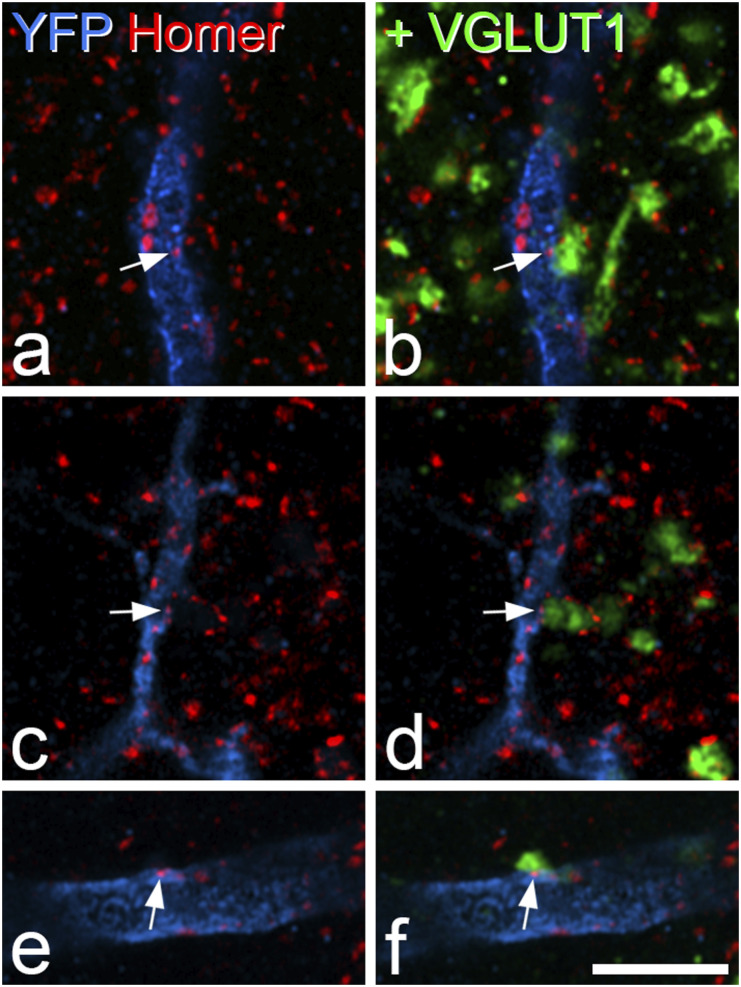
Contacts on deep dorsal horn Phox2a cells from VGLUT1-immunoreactive boutons. (a, b) Part of a dendrite of an antenna cell from a Phox2a::Cre;Ai32 mouse labelled to reveal YFP (blue), Homer (red) and VGLUT1 (green). The dendrite contains several Homer puncta, one of which is marked by an arrow. This punctum is associated with a VGLUT1 bouton that contacts several other Homer puncta, and is therefore likely to originate from a myelinated low-threshold mechanoreceptive (A-LTMR) primary afferent. (c, d) Part of a dendrite belonging to a non-antenna Phox2a cell that was located in the deep part of the dorsal horn. Again, a Homer punctum (arrow) in the dendrite is associated with a VGLUT1 bouton that contacts several other Homer puncta. (e, f) A dendrite from a different non-antenna deep dorsal horn Phox2a cell has a Homer punctum (arrow) that is contacted by a small VGLUT1 profile. This profile is not associated with multiple Homer puncta, and is therefore likely to originate from a corticospinal axon. Images are projections of 2 (a, b, e, f) or 3 (c, d) confocal optical sections at 0.5 μm z-spacing. Scale bar = 5 μm. YFP, yellow fluorescent protein.

For the analysis of VGLUT1 contacts onto dendrites of antenna cells, we examined between 1016–2271 (mean 1482) μm length of dendrites belonging to these cells in three mice and identified an average of 21 Homer puncta on shafts and 11.3 on spines that were in contact with a VGLUT1-immunoreactive profile (Figure 6(a) and (b)). The mean density of these VGLUT1-associated Homer puncta on dendritic shafts was 1.7 per 100 μm, while on dendritic spines it was 1.2 per 100 μm (Table 5). We classified VGLUT1-immunoreactive profiles as putative corticospinal (if they were adjacent to only one Homer punctum) or A-LTMR (if they were associated with more than one punctum) and on this basis, we estimate that 56% of the VGLUT1 synapses were from A-LTMRs.

**Table 5.** Quantitative analysis of VGLUT1 input to antenna and non-antenna cells.

	Dendritic shafts		Dendritic spines		% putative A-LTMR
	Number of VGLUT1 contacts	Density of VGLUT1 contacts/100 μm	Number of VGLUT1 contacts	Density of VGLUT1 contacts/100 μm	
Antenna (*n* = 3 mice)	21 (9–30)	1.7 (0.8–2.8)	11.3 (4–17)	1.2 (0.5–2.2)	55.9 (48.6–61.5)
Non-antenna (*n* = 18 cells)	14.6 (3–27)	1.8 (0.6–4.3)	6.1 (0–24)	0.7 (0–2.3)	48.3 (20–66.7)

Note that for the antenna cells, lengths of dendrite were pooled for each mouse, while for the non-antenna cells, six cells from each of the three mice were examined.

**Figure 7.**
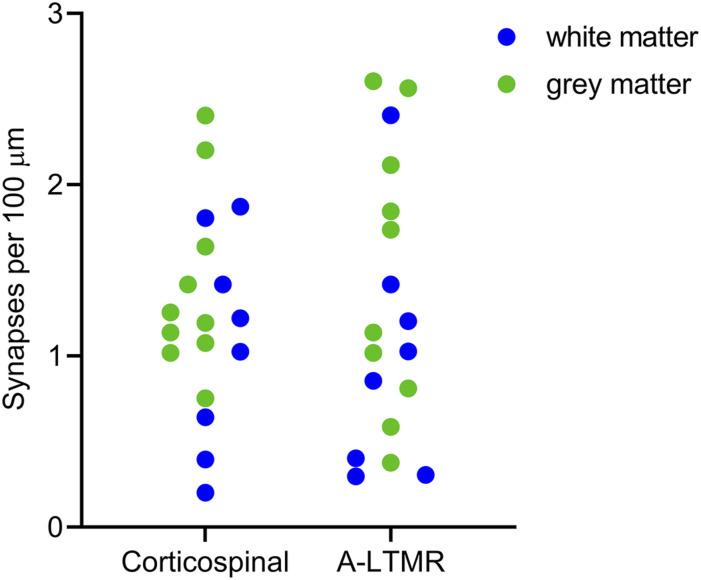
Density of synapses from VGLUT1 profiles on non-antenna cells. Scatter plot showing the density of contacts onto Homer puncta on spines/shafts of non-antenna cells at which a VGLUT1-immunoreactive bouton was present. The cells were located either in the deep grey matter (*n* = 10, green circles) or in the lateral white matter (*n* = 8, blue circles). VGLUT1 boutons contacting these cells were classified as corticospinal if they were only adjacent to one Homer punctum, and as A-low threshold mechanoreceptor (A-LTMR) if they were adjacent to two or more Homer puncta.

VGLUT1 contacts were assessed on 18 non-antenna cells located either in the deep grey matter (laminae IV-V, *n* = 10) or in the lateral white matter (*n* = 8) (Figure 6(c)–(f)). Because we found no significant difference between the pattern of VGLUT1 contacts on cells in these two regions (see below), we pooled the data for the two cell types in Table 5. We examined a mean length of 796 μm (range 328–1171 μm) of dendrite on these cells, and found that the density of VGLUT1 contacts that were adjacent to a Homer punctum on the dendritic shafts averaged 1.8 per 100 μm, while the density of spines with a Homer punctum contacted by a VGLUT1 bouton averaged 0.7 per 100 μm (Table 5). Using the same criteria as those described above, we found that A-LTMRs accounted for just under 50% of the VGLUT1 contacts on the non-antenna cells (Table 5). During the course of this analysis, we noted that the density of VGLUT1 inputs was highly variable between the cells (Figure 7). This was not related to location, since 2-way ANOVA revealed no significant difference in the density of contacts on cells in grey versus white matter (*p* = 0.183), no difference in the density of contacts from the two types of VGLUT1 bouton (*p* = 0.9558), and no interaction between these (*p* = 0.5272).

### Expression of Tac1 by antenna cells

**Figure 8.**
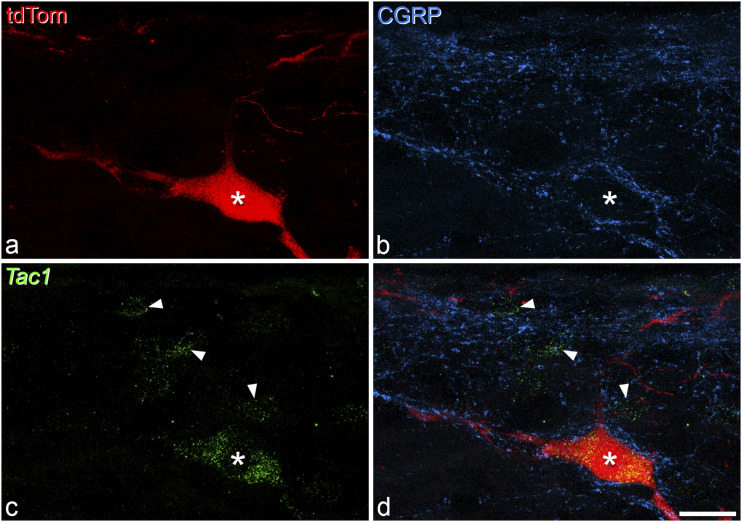
Expression of Tac1 mRNA by an antenna cell. (a) Immunostaining for tdTomato (tdTom) in a Phox2a cell (asterisk) in lamina III. (b) The cell is associated with numerous boutons that are immunoreactive for CGRP (blue), confirming that it is an antenna cell. (c) fluorescent in situ hybridisation for *Tac1* mRNA reveals strong labelling of the antenna cell, and also the presence of *Tac1* in three other cells nearby (marked with arrowheads). (d) A merged image showing expression of *Tac1* in the tdTomato cell. All images are projections of 11 confocal optical sections at 1 μm z-spacing. Scale bar = 20 μm. CGRP, calcitonin gene-related peptide.

We previously showed that intraspinal injection of AAV coding for Cre-dependent GFP into Tac1^Cre^ mice labelled some large cells in laminae III-IV that had dorsal dendrites extending into the SDH,^26^ suggesting that at least some of the antenna-type ALS cells express Tac1. We therefore used a combined in situ hybridisation and immunohistochemistry technique to visualise mRNA for *Tac1* (the gene for substance P) within these cells. TdTomato-positive antenna cells in laminae III-IV were again identified by the presence of numerous contacts from CGRP immunoreactive axons. In this way, we identified 29 antenna cells in the dorsal horns of two mice. *Tac1* mRNA was present in 80% (75–85%) of these cells (11 out of 13 cells in one animal and 12 out of 16 cells in the other) (Figure 8).

## Discussion

The main findings of this study are that the Phox2a::Cre line captures two distinct populations of projection neurons in the deep dorsal horn/lateral white matter, antenna and “non-antenna” cells, and that these differ in their pattern of excitatory synaptic input.

### Antenna cells

Antenna cells form a distinctive population of ALS projection neurons that have been extensively characterized in the rat, in which they can be recognised by expression of the NK1r.^3,7,8,42–47^ All cells of this type were shown to respond to noxious heat, pinch and subcutaneous injection of formaldehyde.^45^

As in the rat,^3^ we found that not all antenna cells were retrogradely labelled from the LPb. However, all of those that were retrogradely labelled were tdTomato positive in the Phox2a::Cre;Ai9 mouse. Although the density of Phox2a cells in lamina I and the lateral white matter varied considerably across the three mice used for quantitative analysis, the density of antenna cells was very consistent, with a mean of 1.77 cells per 60 μm section. Assuming a segment length of 1.5 mm,^49^ this would correspond to ∼44 cells (22 per side) in the segment, and this is similar to the number of NK1r-immunoreactive lamina III-IV projection neurons in the L4 segment of the rat (15–24).^3^ Together, these findings suggest that most, if not all, of the antenna cells are captured by the Phox2a::Cre line. This contrasts with ALS neurons in lamina I, the LSN and deeper laminae, since many of these are not Phox2a-positive.^5,25^

We previously reported that in the rat these cells received between 13 and 19 contacts per 100 μm from CGRP-immunoreactive boutons on their dorsal dendritic shafts.^7^ However, we were not able to determine the proportion of excitatory synapses that these accounted for. Here we show that antenna cells in the mouse receive at least 20 excitatory synapses on shafts, as well as ∼11 on dendritic spines, per 100 μm of dendritic length, and that ∼two-thirds of these are from CGRP-expressing axons. This suggests that the extent of input from peptidergic nociceptors is similar in the two species, and that it accounts for the majority of excitatory synapses on these cells. This represents a highly selective innervation, particularly since much of the dendritic trees of these cells lies outside the plexus of CGRP axons in laminae I-IIo.

Additional sources of excitatory input to the antenna cells could include other classes of primary afferent, glutamatergic spinal neurons and axons descending from the brain. In the rat, antenna cells were shown to receive sparse contacts from A-LTMRs.^44^ Here we found that the density of VGLUT1 synapses per 100 μm of dendrite on these cells was 1.7 for dendritic shafts and 1.2 for dendritic spines. The overall densities of Homer puncta on shafts and spines of these cells were 19.9 and 11 per 100 μm, respectively (Table 3). However, since we only examined VGLUT1 contacts in laminae IIi-V, we recalculated these values for dendrites of the antenna cells that lay within this region. This gave densities of 19 and 11.2 per 100 μm for Homer puncta on shafts and spines. Based on this, we estimate that VGLUT1 boutons account for ∼10% of the excitatory synapses on dendrites below lamina IIo. Around half of these boutons were associated with two or more Homer puncta, and are likely to be derived from A-LTMRs,^33–36^ while the remainder probably belong to corticospinal tract axons.^32^ As noted above, glutamatergic spinal neurons generally express high levels of VGLUT2, and axons of these cells would be classified as CGRP-negative and strongly immunoreactive for VGLUT2.^28,50,51^ Only ∼5% of the Homer puncta on the antenna cells were associated with boutons of this type, suggesting that antenna cells receive little input from either local glutamatergic interneurons or collateral axons of dorsal horn projection neurons.^52^ We had previously reported that antenna cells in rat received numerous contacts from boutons with strong VGLUT2 immunoreactivity.^53^ However, although we found many contacts from axons of this type in the present study, most were not associated with Homer puncta on the antenna cell, suggesting that they were not targeting these cells. Around 7% of the excitatory synapses on the antenna cells were associated with CGRP-negative boutons that showed weak VGLUT2-immunostaining, and these could include A-LTMRs, as these may express low levels of VGLUT2.^28,54^ We previously reported that antenna cells in the rat had very few contacts from axons that bound the lectin IB4, and it is therefore unlikely that these cells receive significant synaptic input from non-peptidergic nociceptors.^46^

Hunt et al.^55^ reported that in rats treated with intraspinal colchicine (to increase the concentration of peptides in cell bodies), SP-containing cells in lamina III often had dorsally directed dendrites that were associated with plexuses of SP-immunoreactive axons. Our finding that most antenna cells possessed *Tac1* mRNA therefore suggests that the cells described by Hunt et al. were ALS projection neurons of the antenna type. Tac1 is also expressed by ∼40% of ALS neurons in lamina I,^50^ as well as by some of those in the LSN.^5,25^ Previous studies have proposed specific roles for Tac1-expressing ALS projection neurons,^56,57^ and our results suggest that these are a heterogeneous population.

It is likely that a similar population of antenna cells exists in primates, since some lamina V spinothalamic tract cells in monkey have dendrites that extend as far as lamina I,^58^ and these cells can receive synapses from CGRP-immunoreactive axons on both their distal dendrites in laminae I-IIo, and also on deeper dendrites in laminae III-V.^59^ In addition, Golgi staining of human spinal cord has revealed “antenna” cells in laminae III and IV with dendrites extending into the SDH,^4^ and it has been shown that the dorsal dendrites of some cells in laminae IV-V are associated with bundles of CGRP-immunoreactive axons.^60^

### “Non-antenna” projection neurons in laminae III-V and the lateral white matter

Numerous electrophysiological studies carried out in vivo in various species have examined the responses of ALS projection neurons in the deep dorsal horn and lateral white matter, and shown that these cells can respond to a range of noxious and innocuous stimuli, with many showing WDR response profiles.^61^ However, these studies seldom involved intracellular labelling and analysis of dendritic trees, which means that recorded cells could not be assigned to the antenna or non-antenna groups described here. Interestingly, Surmeier et al.^62^ identified four classes of spinothalamic neurons in laminae IV-V of the monkey. One of these groups (A) accounted for 55% of recorded cells, and consisted of cells that responded weakly to a range of cutaneous stimuli, while another class (D) constituted 29% of the recorded cells and these responded strongly to noxious stimuli. The two remaining classes (B and C) showed strong responses to low- or intermediate-intensity stimuli, respectively. Reconstruction of dendritic trees of representative cells showed that those in class D had profuse dendritic branching in laminae I-II, and these may therefore correspond to the antenna cells. Cells in class A had predominantly ventral dendrites, while those in classes B and C preferentially arborised in lamina III. If this interpretation is correct, then cells in classes A-C would be included in the non-antenna populations that we describe here.

Based on these response properties, projection neurons in this region must receive input, either directly or indirectly, from various primary afferent classes, including nociceptors and LTMRs. Electrophysiological studies have revealed monosynaptic input to primate spinothalamic cells in deep dorsal horn from primary afferents that are likely to correspond to A-LTMRs,^63^ while anatomical tracing from dorsal roots combined with retrograde labelling of spinothalamic tract cells in the rat has demonstrated contacts from primary afferents onto cell bodies and proximal dendrites of these cells.^64^

We found that the mean densities of Homer puncta on dendritic shafts and spines of the deep non-antenna cells were 14.2 and 4.3/100 μm, respectively, while the corresponding mean values for the density of VGLUT1 contacts on these puncta were 1.8 for shafts and 0.7 for spines. This suggests that on average, around 12% of the excitatory synapses on these cells are from VGLUT1-expressing axons, with approximately equal numbers originating from A-LTMRs and corticospinal axons. However, it was clear that the extent of VGLUT1 input of both types was highly variable, suggesting that some cells will receive a far higher proportion of their input from A-LTMR and/or corticospinal boutons, while for others the proportion will be much lower. This variability is likely to reflect the heterogeneity of classes A-C defined by Surmeier et al.^62^

At least part of the nociceptive drive to these cells results from monosynaptic input from peptidergic afferents, since all six of the cells analysed received some synapses from CGRP boutons. However, since their dendrites do not reach the superficial laminae, where most nociceptive afferents terminate, much of this drive is likely to be relayed through excitatory interneurons located in the SDH.^65^ Consistent with this, we found that all of the cells received numerous synapses from boutons with strong VGLUT2 expression, which are likely to originate mainly from dorsal horn interneurons. The cells giving rise to this input have not yet been identified, but we recently reported that GRPR-expressing excitatory interneurons in the SDH, which correspond to a morphologically-defined class known as vertical cells, give rise to axons that arborise locally as well as projecting to the lateral part of lamina V and the lateral white matter.^66^ The GRPR cells were shown to respond to both noxious and pruritic stimuli, and may therefore relay this information to the deep non-antenna projection cells.

## Conclusion

Our results show that antenna cells are effectively captured in the Phox2a::Cre mouse line, and that these form a distinctive subset of deep Phox2a cells. These cells are homogeneous in terms of their morphology and synaptic inputs, suggesting that they have a specific role in somatosensation. In contrast, the non-antenna cells appear to be far more heterogeneous. Future studies with single-cell RNAsequencing may help to reveal distinct classes among these cells, and this will be a pre-requisite for investigating their synaptic connectivity and function.

## Appendix

### Abbreviations

A-LTMR: A-low threshold mechanoreceptor
ALS: anterolateral system
CGRP: calcitonin gene-related peptide
CTb: cholera toxin B subunit
GFP: green fluorescent protein
GRPR: gastrin releasing peptide receptor
LPb: lateral parabrachial area
LSN: lateral spinal nucleus
NK1r: neurokinin 1 receptor
NPY: neuropeptide Y
PBS: phosphate buffered saline
SDH: superficial dorsal horn
SP: substance P
WDR: wide dynamic range
YFP: yellow fluorescent protein

## References

[1] Wercberger R Basbaum AI . Spinal cord projection neurons: a superficial, and also deep, analysis. Curr Opin Physiol 2019; 11: 109–115. 32864531 10.1016/j.cophys.2019.10.002 PMC7450810

[2] Willis WD Coggeshall RE . Sensory mechanisms of the spinal cord. New York, NY: John Wiley, 1991.

[3] Todd AJ McGill MM Shehab SA . Neurokinin 1 receptor expression by neurons in laminae I, III and IV of the rat spinal dorsal horn that project to the brainstem. Eur J Neurosci 2000; 12: 689–700. 10712649 10.1046/j.1460-9568.2000.00950.x

[4] Schoenen J . The dendritic organization of the human spinal cord: the dorsal horn. Neuroscience 1982; 7: 2057–2087. 7145088 10.1016/0306-4522(82)90120-8

[5] Roome RB Bourojeni FB Mona B Rastegar-Pouyani S Blain R Dumouchel A Salesse C Thompson WS Brookbank M Gitton Y Tessarollo L Goulding M Johnson JE Kmita M Chédotal A Kania A Phox2a defines a developmental origin of the anterolateral system in mice and humans. Cell Rep 2020; 33: 108425. 33238113 10.1016/j.celrep.2020.108425 PMC7713706

[6] Cameron D Polgar E Gutierrez-Mecinas M Gomez-Lima M Watanabe M Todd AJ . The organisation of spinoparabrachial neurons in the mouse. Pain 2015; 156: 2061–2071. 26101837 10.1097/j.pain.0000000000000270 PMC4770364

[7] Naim M Spike RC Watt C Shehab SA Todd AJ . Cells in laminae III and IV of the rat spinal cord that possess the neurokinin-1 receptor and have dorsally directed dendrites receive a major synaptic input from tachykinin-containing primary afferents. J Neurosci 1997; 17: 5536–5548. 9204935 10.1523/JNEUROSCI.17-14-05536.1997 PMC6793839

[8] Polgár E Shehab SA Watt C Todd AJ . GABAergic neurons that contain neuropeptide Y selectively target cells with the neurokinin 1 receptor in laminae III and IV of the rat spinal cord. J Neurosci 1999; 19: 2637–2646. 10087077 10.1523/JNEUROSCI.19-07-02637.1999 PMC6786068

[9] Spike RC Puskar Z Andrew D Todd AJ . A quantitative and morphological study of projection neurons in lamina I of the rat lumbar spinal cord. Eur J Neurosci 2003; 18: 2433–2448. 14622144 10.1046/j.1460-9568.2003.02981.x

[10] Lahuerta J Bowsher D Campbell J Lipton S . Clinical and instrumental evaluation of sensory function before and after percutaneous anterolateral cordotomy at cervical level in man. Pain 1990; 42: 23–30. 1700355 10.1016/0304-3959(90)91087-Y

[11] Marshall AG Sharma ML Marley K Olausson H McGlone FP . Spinal signalling of C-fiber mediated pleasant touch in humans. Elife 2019; 8: e51642. 31872799 10.7554/eLife.51642 PMC6964968

[12] Bernard JF Dallel R Raboisson P Villanueva L Le Bars D . Organization of the efferent projections from the spinal cervical enlargement to the parabrachial area and periaqueductal gray: a PHA-L study in the rat. J Comp Neurol 1995; 353: 480–505. 7759612 10.1002/cne.903530403

[13] Feil K Herbert H . Topographic organization of spinal and trigeminal somatosensory pathways to the rat parabrachial and Kolliker-Fuse nuclei. J Comp Neurol 1995; 353: 506–528. 7759613 10.1002/cne.903530404

[14] Gauriau C Bernard JF . A comparative reappraisal of projections from the superficial laminae of the dorsal horn in the rat: the forebrain. J Comp Neurol 2004; 468: 24–56. 14648689 10.1002/cne.10873

[15] Allard J . Physiological properties of the lamina I spinoparabrachial neurons in the mouse. J Physiol 2019; 597: 2097–2113. 30719699 10.1113/JP277447 PMC6441934

[16] Bester H Chapman V Besson JM Bernard JF . Physiological properties of the lamina I spinoparabrachial neurons in the rat. J Neurophysiol 2000; 83: 2239–2259. 10758132 10.1152/jn.2000.83.4.2239

[17] Hachisuka J Koerber HR Ross SE . Selective-cold output through a distinct subset of lamina I spinoparabrachial neurons. Pain 2020; 161: 185–194. 31577643 10.1097/j.pain.0000000000001710 PMC10461608

[18] Ferrington DG Sorkin LS Willis WD Jr . Responses of spinothalamic tract cells in the superficial dorsal horn of the primate lumbar spinal cord. J Physiol 1987; 388: 681–703. 3656204 10.1113/jphysiol.1987.sp016638 PMC1192572

[19] Andrew D . Sensitization of lamina I spinoparabrachial neurons parallels heat hyperalgesia in the chronic constriction injury model of neuropathic pain. J Physiol 2009; 587: 2005–2017. 19289544 10.1113/jphysiol.2009.170290 PMC2689339

[20] Menetrey D Chaouch A Besson JM . Location and properties of dorsal horn neurons at origin of spinoreticular tract in lumbar enlargement of the rat. J Neurophysiol 1980; 44: 862–877. 7441321 10.1152/jn.1980.44.5.862

[21] Menetrey D de Pommery J Besson JM . Electrophysiological characteristics of lumbar spinal cord neurons backfired from lateral reticular nucleus in the rat. J Neurophysiol 1984; 52: 595–611. 6491707 10.1152/jn.1984.52.4.595

[22] Fields HL Clanton CH Anderson SD . Somatosensory properties of spinoreticular neurons in the cat. Brain Res 1977; 120: 49–66. 832119 10.1016/0006-8993(77)90497-8

[23] Giesler GJ Jr Yezierski RP Gerhart KD Willis WD . Spinothalamic tract neurons that project to medial and/or lateral thalamic nuclei: evidence for a physiologically novel population of spinal cord neurons. J Neurophysiol 1981; 46: 1285–1308. 7320746 10.1152/jn.1981.46.6.1285

[24] Davidson S Zhang X Khasabov SG Moser HR Honda CN Simone DA Giesler GJ Jr . Pruriceptive spinothalamic tract neurons: physiological properties and projection targets in the primate. J Neurophysiol 2012; 108: 1711–1723. 22723676 10.1152/jn.00206.2012 PMC3544948

[25] Alsulaiman WAA Quillet R Bell AM Dickie AC Polgar E Boyle KA Watanabe M Roome RB Kania A Todd AJ Gutierrez-Mecinas M . Characterisation of lamina I anterolateral system neurons that express Cre in a Phox2a-Cre mouse line. Sci Rep 2021; 11: 17912. 34504158 10.1038/s41598-021-97105-w PMC8429737

[26] Gutierrez-Mecinas M Bell AM Marin A Taylor R Boyle KA Furuta T Watanabe M Polgár E Todd AJ . Preprotachykinin A is expressed by a distinct population of excitatory neurons in the mouse superficial spinal dorsal horn including cells that respond to noxious and pruritic stimuli. Pain 2017; 158: 440–456. 27902570 10.1097/j.pain.0000000000000778 PMC5302415

[27] Gutierrez-Mecinas M Kuehn ED Abraira VE Polgár E Watanabe M Todd AJ . Immunostaining for Homer reveals the majority of excitatory synapses in laminae I-III of the mouse spinal dorsal horn. Neuroscience 2016; 329: 171–181. 27185486 10.1016/j.neuroscience.2016.05.009 PMC4915440

[28] Todd AJ Hughes DI Polgár E Nagy GG Mackie M Ottersen OP Maxwell DJ . The expression of vesicular glutamate transporters VGLUT1 and VGLUT2 in neurochemically defined axonal populations in the rat spinal cord with emphasis on the dorsal horn. Eur J Neurosci 2003; 17: 13–27. 12534965 10.1046/j.1460-9568.2003.02406.x

[29] Alvarez FJ Villalba RM Zerda R Schneider SP . Vesicular glutamate transporters in the spinal cord, with special reference to sensory primary afferent synapses. J Comp Neurol 2004; 472: 257–280. 15065123 10.1002/cne.20012

[30] Oliveira AL Hydling F Olsson E Shi T Edwards RH Fujiyama F Kaneko T Hokfelt T Cullheim S Meister B . Cellular localization of three vesicular glutamate transporter mRNAs and proteins in rat spinal cord and dorsal root ganglia. Synapse 2003; 50: 117–129. 12923814 10.1002/syn.10249

[31] Landry M Bouali-Benazzouz R El Mestikawy S Ravassard P Nagy F . Expression of vesicular glutamate transporters in rat lumbar spinal cord, with a note on dorsal root ganglia. J Comp Neurol 2004; 468: 380–394. 14681932 10.1002/cne.10988

[32] Abraira VE Kuehn ED Chirila AM Springel MW Toliver AA Zimmerman AL Orefice LL Boyle KA Bai L Song BJ Bashista KA O’Neill TG Zhuo J Tsan C Hoynoski SM Nelson SB Heintz N Hughes DI Ginty DD The cellular and synaptic architecture of the mechanosensory dorsal horn. Cell 2017; 168: 295–310. 28041852 10.1016/j.cell.2016.12.010 PMC5236062

[33] Nagy GG Al-Ayyan M Andrew D Fukaya M Watanabe M Todd AJ . Widespread expression of the AMPA receptor GluR2 subunit at glutamatergic synapses in the rat spinal cord and phosphorylation of GluR1 in response to noxious stimulation revealed with an antigen-unmasking method. J Neurosci 2004; 24: 5766–5777. 15215299 10.1523/JNEUROSCI.1237-04.2004 PMC6729210

[34] Maxwell DJ Rethelyi M . Ultrastructure and synaptic connections of cutaneous afferent fibres in the spinal cord. Trends Neurosci 1987; 10: 117–123.

[35] Rethelyi M Light AR Perl ER . Synaptic complexes formed by functionally defined primary afferent units with fine myelinated fibers. J Comp Neurol 1982; 207: 381–393. 6288776 10.1002/cne.902070409

[36] Ribeiro-da-Silva A Coimbra A . Two types of synaptic glomeruli and their distribution in laminae I-III of the rat spinal cord. J Comp Neurol 1982; 209: 176–186. 6890076 10.1002/cne.902090205

[37] Choi HMT Schwarzkopf M Fornace ME Acharya A Artavanis G Stegmaier J Cunha A Pierce NA . Third-generation in situ hybridization chain reaction: multiplexed, quantitative, sensitive, versatile, robust. Development 2018; 145: 165753. 10.1242/dev.165753 PMC6031405 29945988

[38] Cuello AC Galfre G Milstein C . Detection of substance P in the central nervous system by a monoclonal antibody. Proc Natl Acad Sci U S A 1979; 76: 3532–3536. 386341 10.1073/pnas.76.7.3532 PMC383862

[39] McLeod AL Krause JE Ribeiro-Da-Silva A . Immunocytochemical localization of neurokinin B in the rat spinal dorsal horn and its association with substance P and GABA: an electron microscopic study. J Comp Neurol 2000; 420: 349–362. 10754507

[40] Rowan S Todd AJ Spike RC . Evidence that neuropeptide Y is present in GABAergic neurons in the superficial dorsal horn of the rat spinal cord. Neuroscience 1993; 53: 537–545. 8492914 10.1016/0306-4522(93)90218-5

[41] Iwagaki N Ganley RP Dickie AC Polgár E Hughes DI Del Rio P Revina Y Watanabe M Todd AJ Riddell JS . A combined electrophysiological and morphological study of neuropeptide Y-expressing inhibitory interneurons in the spinal dorsal horn of the mouse. Pain 2016; 157: 598–612. 26882346 10.1097/j.pain.0000000000000407 PMC4751741

[42] Bleazard L Hill RG Morris R . The correlation between the distribution of the NK1 receptor and the actions of tachykinin agonists in the dorsal horn of the rat indicates that substance P does not have a functional role on substantia gelatinosa (lamina II) neurons. J Neurosci 1994; 14: 7655–7664. 7527847 10.1523/JNEUROSCI.14-12-07655.1994 PMC6576917

[43] Brown JL Liu H Maggio JE Vigna SR Mantyh PW Basbaum AI . Morphological characterization of substance P receptor-immunoreactive neurons in the rat spinal cord and trigeminal nucleus caudalis. J Comp Neurol 1995; 356: 327–344. 7642798 10.1002/cne.903560302

[44] Naim MM Shehab SA Todd AJ . Cells in laminae III and IV of the rat spinal cord which possess the neurokinin-1 receptor receive monosynaptic input from myelinated primary afferents. Eur J Neurosci 1998; 10: 3012–3019. 9758171 10.1111/j.1460-9568.1998.00335.x

[45] Polgár E Campbell AD MacIntyre LM Watanabe M Todd AJ . Phosphorylation of ERK in neurokinin 1 receptor-expressing neurons in laminae III and IV of the rat spinal dorsal horn following noxious stimulation. Mol Pain 2007; 3: 4. 17309799 10.1186/1744-8069-3-4 PMC1803781

[46] Sakamoto H Spike RC Todd AJ . Neurons in laminae III and IV of the rat spinal cord with the neurokinin-1 receptor receive few contacts from unmyelinated primary afferents which do not contain substance P. Neuroscience 1999; 94: 903–908. 10579582 10.1016/s0306-4522(99)00346-2

[47] Marshall GE Shehab SA Spike RC Todd AJ . Neurokinin-1 receptors on lumbar spinothalamic neurons in the rat. Neuroscience 1996; 72: 255–263. 8730722 10.1016/0306-4522(95)00558-7

[48] Szentagothai J Rethelyi M . Cyto- and neuropil architecture of the spinal cord. In: Desmedt JE (ed) New developments in electromyography and clinical neurophysiol. Basel, Switzerland: Karger, 1973, Vol. 3, pp. 20–37.

[49] Polgár E Durrieux C Hughes DI Todd AJ . A quantitative study of inhibitory interneurons in laminae I-III of the mouse spinal dorsal horn. PLoS One 2013; 8: e78309. 24205193 10.1371/journal.pone.0078309 PMC3808353

[50] Polgar E Bell AM Gutierrez-Mecinas M Dickie AC Akar O Costreie M Watanabe M Todd AJ . Substance P-expressing neurons in the superficial dorsal horn of the mouse spinal cord: insights into their functions and their roles in synaptic circuits. Neuroscience 2020; 450: 113–125. 32634530 10.1016/j.neuroscience.2020.06.038 PMC7717171

[51] Bell AM Gutierrez-Mecinas M Stevenson A Casas-Benito A Wildner H West SJ Watanabe M Todd AJ . Expression of green fluorescent protein defines a specific population of lamina II excitatory interneurons in the GRP::eGFP mouse. Sci Rep 2020; 10: 13176. 32764601 10.1038/s41598-020-69711-7 PMC7411045

[52] Szucs P Luz LL Lima D Safronov BV . Local axon collaterals of lamina I projection neurons in the spinal cord of young rats. J Comp Neurol 2010; 518: 2645–2665. 20506469 10.1002/cne.22391

[53] Baseer N Polgar E Watanabe M Furuta T Kaneko T Todd AJ . Projection neurons in lamina III of the rat spinal cord are selectively innervated by local dynorphin-containing excitatory neurons. J Neurosci 2012; 32: 11854–11863. 22915126 10.1523/JNEUROSCI.2707-12.2012 PMC3438856

[54] Persson S Boulland JL Aspling M Larsson M Fremeau RT Jr Edwards RH Storm-Mathisen J Chaudhry FA Broman J . Distribution of vesicular glutamate transporters 1 and 2 in the rat spinal cord, with a note on the spinocervical tract. J Comp Neurol 2006; 497: 683–701. 16786558 10.1002/cne.20987

[55] Hunt SP Kelly JS Emson PC Kimmel JR Miller RJ Wu JY . An immunohistochemical study of neuronal populations containing neuropeptides or gamma-aminobutyrate within the superficial layers of the rat dorsal horn. Neuroscience 1981; 6: 1883–1898. 6170908 10.1016/0306-4522(81)90029-4

[56] Huang T Lin SH Malewicz NM Zhang Y Zhang Y Goulding M LaMotte RH Ma Q . Identifying the pathways required for coping behaviours associated with sustained pain. Nature 2019; 565: 86–90. 30532001 10.1038/s41586-018-0793-8 PMC6461409

[57] Barik A Sathyamurthy A Thompson J Seltzer M Levine A Chesler A . A spinoparabrachial circuit defined by Tacr1 expression drives pain. Elife 2021; 10: e61135. 33591273 10.7554/eLife.61135 PMC7993995

[58] Carlton SM Lamotte CC Honda CN Surmeier DJ Delanerolle N Willis WD . Ultrastructural analysis of axosomatic contacts on functionally identified primate spinothalamic tract neurons. J Comp Neurol 1989; 281: 555–566. 2708581 10.1002/cne.902810406

[59] Carlton SM Westlund KN Zhang DX Sorkin LS Willis WD . Calcitonin gene-related peptide containing primary afferent fibers synapse on primate spinothalamic tract cells. Neurosci Lett 1990; 109: 76–81. 2314643 10.1016/0304-3940(90)90540-p

[60] Jakab G Salamon I Petrusz P Rethelyi M . Termination patterns of calcitonin gene-related peptide-immunoreactive nerve fibers in the dorsal horn of the human spinal cord. Exp Brain Res 1990; 80: 609–617. 2387358 10.1007/BF00228000

[61] Willis WD Coggeshall RE . Sensory mechanisms of the spinal cord. New York, NY: Kluwer Academic, 2004, Vol. 1.

[62] Surmeier DJ Honda CN Willis WD Jr . Natural groupings of primate spinothalamic neurons based on cutaneous stimulation. Physiological and anatomical features. J Neurophysiol 1988; 59: 833–860. 3367200 10.1152/jn.1988.59.3.833

[63] Foreman RD Applebaum AE Beall JE Trevino DL Willis WD . Responses of primate spinothalamic tract neurons to electrical stimulation of hindlimb peripheral nerves. J Neurophysiol 1975; 38: 132–145. 162940 10.1152/jn.1975.38.1.132

[64] Bolton PS Tracey DJ . Spinothalamic and propriospinal neurones in the upper cervical cord of the rat: terminations of primary afferent fibres on soma and primary dendrites. Exp Brain Res 1992; 92: 59–68. 1486955 10.1007/BF00230383

[65] Light AR Kavookjian AM . Morphology and ultrastructure of physiologically identified substantia gelatinosa (lamina II) neurons with axons that terminate in deeper dorsal horn laminae (III-V). J Comp Neurol 1988; 267: 172–189. 3343395 10.1002/cne.902670203

[66] Polgár E Dickie AC Gutierrez-Mecinas M Bell AM Boyle KA Quillet R Ab Rashid E Clark RA German MT Watanabe M Riddell JS Todd AJ . Grpr expression defines a population of superficial dorsal horn vertical cells that have a role in both itch and pain. Pain 2022. in press. 10.1097/j.pain.0000000000002677 PMC9756441 35543635

